# Admission uric acid to HDL-C ratio predicts 90-day post-stroke depression in acute ischemic stroke

**DOI:** 10.3389/fpsyt.2025.1693156

**Published:** 2025-11-03

**Authors:** Xiangqi Kong, Xinyue Yuan, Haobo Wang, Mina Zhao, Wei Jing

**Affiliations:** Third Hospital of Shanxi Medical University, Shanxi Bethune Hospital, Shanxi Academy of Medical Sciences, Tongji Shanxi Hospital, Taiyuan, China

**Keywords:** PSD, UHR, biomarker, AIS, prognosis

## Abstract

**Background:**

Post-stroke depression (PSD) affects 30% of acute ischemic stroke (AIS) survivors, compromising functional recovery and increasing mortality. Uric acid (UA) and high-density lipoprotein cholesterol (HDL) modulate oxidative stress and neuroinflammation, but their individual prognostic value is inconsistent. This study investigated whether the admission UA/HDL ratio (UHR) independently predicts 90-day PSD in AIS patients.

**Methods:**

We retrospectively analyzed 541 AIS patients admitted to Shanxi Bethune Hospital (October 2023–December 2024). Inclusion required first-ever AIS confirmed by CT or MRI within 72 hours. Demographics, clinical variables, and laboratory data were collected. Multivariable logistic regression, subgroup analyses, and restricted cubic spline models evaluated associations between UHR and PSD, adjusting for confounders.

**Results:**

Of 541 patients, 193 (35.7%) developed PSD. PSD patients had higher UHR, elevated NIHSS scores, reduced neutrophil counts, and lower cognitive scores (all p<0.05). UHR independently predicted PSD (adjusted OR per 1-unit increase: 1.0023 and per 1-SD increase: 1.4725; p=0.0042). Patients in the highest UHR quartile had a 2.17-fold higher PSD risk versus the lowest quartile (p=0.044), with a significant linear dose–response (p=0.013). Subgroup analyses confirmed consistent associations across stroke severity, sex, and comorbidities.

**Conclusions:**

Admission UA/HDL ratio is a robust, independent predictor of 90-day PSD in AIS patients. As an accessible, cost-effective marker, UHR may enable early identification of high-risk individuals during the acute post-stroke phase.

## 1 Introduction

Post-stroke depression (PSD) is a frequent neuropsychiatric complication of acute ischemic stroke (AIS), affecting nearly one-third of survivors (1). In the multicenter cohort study in China, the 90-day cumulative incidence of PSD reached 36.4% (2). PSD exerts multiple adverse effects on patient outcomes. It is associated with poor rehabilitation, recurrent vascular events, reduced quality of life, and a markedly increased mortality risk (3). Previous studies have reported that PSD increases the 5-year mortality risk by approximately 59% (4). Therefore, early identification of patients at high risk for PSD is crucial for reducing post-stroke complications and improving functional outcomes. However, the lack of early, accessible, and integrative biomarkers limits the ability to identify high-risk patients after AIS, thereby constraining the effectiveness of early interventions for PSD.

Addressing this clinical challenge requires exploring potential biomarkers through the underlying pathological mechanisms. Current evidence indicates that the oxidative stress–neuroinflammation cascade induced by acute ischemia constitutes the central biological basis of PSD (5). Within this pathological process, the role of uric acid (UA) remains highly debated. As the end product of purine metabolism, UA exhibits dual properties: it functions as an antioxidant at physiological levels by scavenging free radicals, but at elevated levels, it promotes oxidative stress and neuroinflammation (6). This dual nature has yielded conflicting results in PSD studies: in elderly stroke patients, higher UA levels at admission were closely associated with severe PSD within three months post-stroke (7). Conversely, another study reported that lower serum UA levels at admission were linked to PSD status at the time of discharge (8). Adding complexity, further research indicates that UA levels (≥328.1 μmol/L and ≤239.0 μmol/L) are independently associated with PSD at three months post-stroke, supporting the concept of a UA “biological range” (9). Recent evidence confirms an inverse nonlinear relationship between UA and PSD risk in AIS patients (10). This complexity complicates the clinical interpretation and application of a single UA measure, as it neither allows for the definition of a safe threshold nor supports a standardized predictive criterion.

High-density lipoprotein cholesterol (HDL) interacts with UA to maintain a dynamic balance along the oxidative–anti-inflammatory axis, yet its clinical significance remains uncertain. Although HDL confers anti-inflammatory and antioxidant effects through reverse cholesterol transport and endothelial function enhancement, and is theoretically a protective marker against PSD, clinical findings remain inconsistent: One study reported that PSD patients had significantly lower HDL levels compared with non-PSD individuals; however, this association lost statistical significance in multivariate regression analysis (11). Conversely, another study identified reduced HDL levels as an independent predictor of PSD (12). These discrepancies reflect not only variations in sample characteristics but also the inherent limitations of relying on a single HDL measure, which is susceptible to external influences such as diet and medication, raising concerns about its stability as a predictor of PSD.

Given the functional antagonism between UA and HDL along the oxidative–antioxidative and pro-inflammatory–anti-inflammatory axes, their ratio (uric acid to HDL ratio, UHR) may more accurately reflect post-stroke metabolic and inflammatory dysregulation. However, current evidence is conflicting. A cross-sectional study in U.S. adults reported a significant positive association between elevated UHR and depression risk (13). Conversely, in middle-aged and older Chinese populations, UHR was significantly inversely associated with depressive symptoms (14). While these discrepancies partly reflect population heterogeneity, a more critical limitation is that all studies were confined to general or elderly populations, with no investigation in the context of AIS, a distinct pathological state. Post-stroke acute inflammatory surges, metabolic disturbances, and neural remodeling may fundamentally modify the interaction between HDL and UA, rendering the predictive value of UHR for PSD substantially different from that observed in the general population. Nevertheless, no studies to date have examined the predictive utility of UHR for PSD in patients after AIS.

In summary, although the inflammation–oxidative stress hypothesis has emerged as the dominant framework in PSD research, current predictive markers are largely restricted to single inflammatory factors or conventional lipid parameters, and comprehensive biomarkers integrating oxidative and antioxidative capacities are lacking. Therefore, this study, for the first time, introduces UHR—an “oxidative–antioxidative integrated index”—into the AIS population, aiming to examine whether elevated UHR at admission is independently associated with PSD at 90 days. This investigation offers a novel perspective for PSD risk assessment, shifting from a “single-dimension” approach to one of “systemic balance,” and holds promise for providing a cost-effective, easily measurable, and mechanism-based PSD risk warning tool during the acute phase of stroke.

## 2 Methods

### 2.1 Participants

A retrospective analysis was conducted on patients diagnosed with acute ischemic stroke in the Department of Neurology at Shanxi Bethune Hospital between October 2023 and December 2024.Patients with ischemic stroke were included based on the World Health Organization (WHO) definition, and all diagnoses were confirmed using magnetic resonance imaging (MRI) or cranial computed tomography (CT).

Inclusion criteria were: (1) stroke confirmed by CT or MRI within 72 hours of onset, in accordance with the 2018 Chinese Guidelines for Diagnosis and Treatment of Acute Ischemic Stroke; (2) first-ever stroke with no history of severe neurological deficits; (3) age over 18 years; and (4) complete clinical data with informed consent provided.

**Figure 1:**
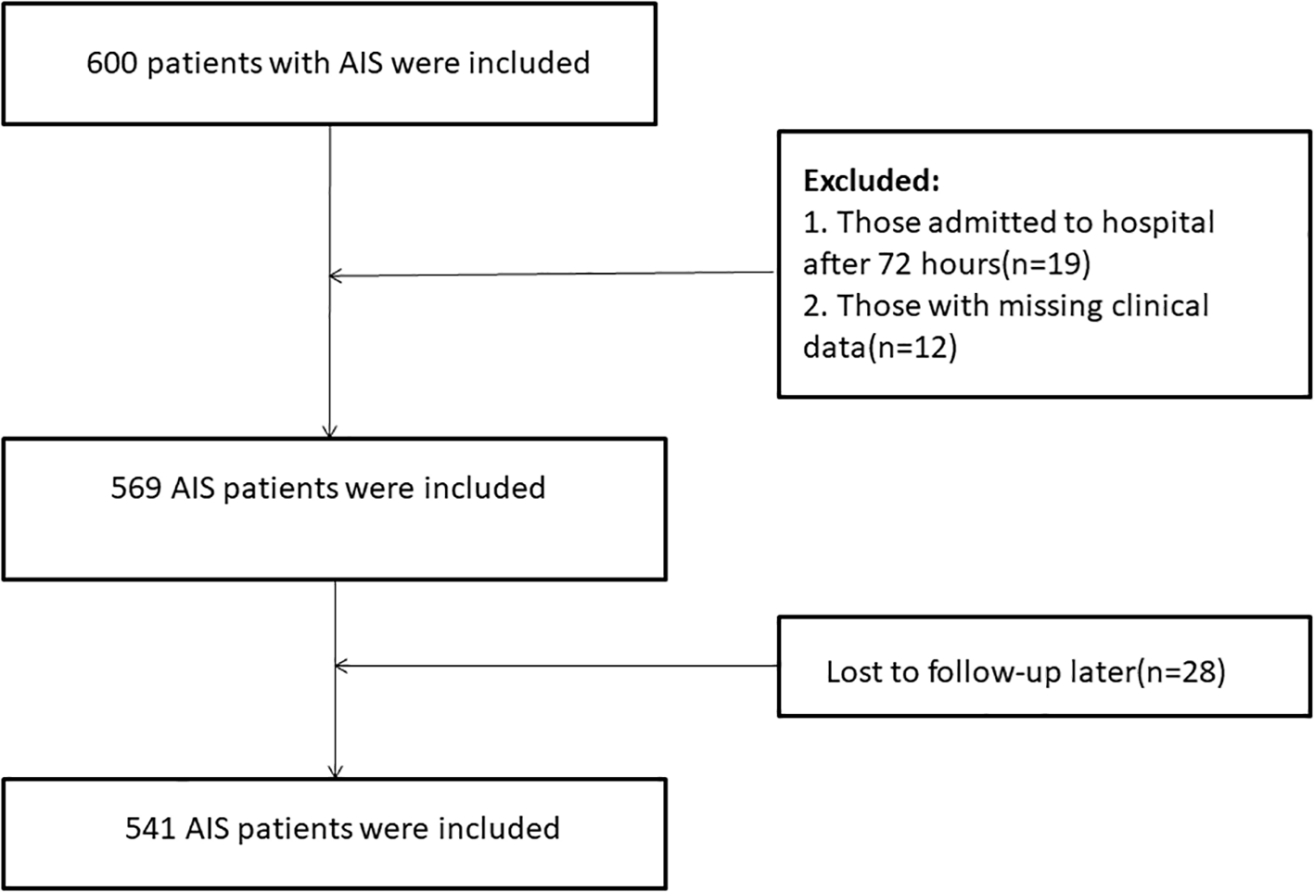
Flowchart of participant selection.

Exclusion criteria were: (1) transient ischemic attack (TIA) or hemorrhagic cerebrovascular disease; (2) any history of psychiatric disorders, either clinically diagnosed or previously treated; (3) severe aphasia or impaired consciousness that prevented participation due to sensory or comprehension limitations; (4) severe cardiopulmonary disease or multi-organ failure; (5) chronic inflammatory conditions; (6) incomplete clinical records; and (7) loss to follow-up. Ultimately, the final analysis comprised 541 eligible participants (**Figure 1**).

### 2.2 Data collection

Patient demographics and clinical characteristics were recorded, including age, sex, education level, responsible lesion, infarct location, history of hypertension (HTN), diabetes mellitus (DM), coronary artery disease (CAD), smoking and alcohol consumption, body mass index (BMI), and National Institutes of Health Stroke Scale (NIHSS) and Montreal Cognitive Assessment (MoCA) scores. Two milliliters of venous blood were collected in EDTA-K2 anticoagulant vacuum tubes, mixed, and analyzed using the fully automated hematology analyzer (Beckman DXH800) to determine white blood cell (WBC), neutrophil (Neu), monocyte (Mon), lymphocyte (Lym), and platelet counts (PLT). Five milliliters of venous blood were collected in serum-separating tubes without anticoagulant and centrifuged to measure triglycerides (TG), total cholesterol (TC), homocysteine (Hcy), uric acid (UA), low-density lipoprotein (LDL), and high-density lipoprotein (HDL). D-dimer levels were measured in anticoagulated plasma prepared with a 1:9 ratio of sodium citrate and analyzed using the Wo fen ACL-TOP750 system. UHR was calculated as uric acid (mmol/L) divided by HDL (mmol/L).

### 2.3 Prognostic assessment

The primary endpoint was the presence of depression 90 days after admission. Two trained clinicians independently administered the Chinese version of the 24-item Hamilton Depression Scale (HAMD-24) while blind to clinical status. Although inter-rater reliability was not re-examined in the present sample, the Chinese HAMD-24 has previously shown excellent internal consistency: the reliability coefficient of the HAMD-24 is 0.99, and that of each item is 0.78–0.98 (15). Patients scoring above 7 on the HAMD were classified as having post-stroke depression (PSD) (16), and this cut-off reference is based on multiple previous Chinese stroke studies that define “presence of depressive symptoms” as a score of ≥ 8 on the HAMD-24 (17, 18). Stroke severity at admission was assessed using NIHSS, with scores <4 indicating mild stroke and scores ≥4 indicating moderate to severe stroke (19). Cognitive function in stroke patients was assessed using MoCA (20).

### 2.4 Statistical analysis

Baseline characteristics were stratified by the presence of post-stroke depression. Continuous variables were tested for normality using the Shapiro-Wilk test and presented as mean ± standard deviation or median (interquartile range, 25th–75th percentile) accordingly. Differences between groups were assessed using the t-test or Mann-Whitney U test. Categorical variables were presented as frequencies and percentages, and group differences were analyzed using the chi-square test.

Multivariable logistic regression was used to evaluate the link between UHR and PSD. Confounding variables identified in univariate analyses were adjusted in multivariable models. Two models were constructed: Model 1 was unadjusted, while Model 2 adjusted for smoking, gender, alcohol consumption, NIHSS, MoCA, Neu, and D-dimer.

In sensitivity analyses, UHR was divided into quartiles as the independent variable, and a linear trend test was conducted to evaluate the robustness of the findings. Restricted cubic spline (RCS) models with four knots were employed to investigate potential nonlinear associations between UHR and post-stroke depression. Furthermore, subgroup analyses and interaction tests were conducted to examine the associations between UHR and adverse outcomes across strata defined by sex, smoking, drinking, HTN, DM, and CAD. Each subgroup analysis was adjusted for the same covariates as in Model 2, excluding the subgroup variable itself. Statistical analyses were performed using R Studio 4.4.2 and DecisonLinnc1.0 software, with p-values <0.05 considered statistically significant.

## 3 Results

### 3.1 Participant baseline characteristics

In total, 541 patients were enrolled in this study, comprising 348 in the non-PSD group and 193 in the PSD group (**Table 1**). Comparisons between groups revealed statistically significant differences in sex, smoking, alcohol consumption, NIHSS score, MoCA, Neu, D-dimer, and UHR (all P < 0.05). Specifically, patients with adverse outcomes were more likely to be male, have a history of smoking and alcohol consumption, exhibit higher NIHSS scores, elevated D-dimer levels and UHR, reduced neutrophil counts, and lower MoCA scores.

**Table 1:** Baselines characteristics of participants.

Characteristic	Overall N = 541^1^	Non-PSD N = 348^1^	PSD N = 193^1^	p-value^2^
Sex				<0.001
Female	162 (30%)	131 (38%)	31 (16%)	
Male	379 (70%)	217 (62%)	162 (84%)	
Education				0.708
illiteracy	88 (16%)	59 (17%)	29 (15%)	
Primary school	193 (36%)	118 (34%)	75 (39%)	
junior high school	218 (40%)	143 (41%)	75 (39%)	
senior high school and above	42 (7.8%)	28 (8.0%)	14 (7.3%)	
Smoking				<0.001
No	220 (41%)	161 (46%)	59 (31%)	
Yes	321 (59%)	187 (54%)	134 (69%)	
Drinking				<0.001
No	269 (50%)	192 (55%)	77 (40%)	
Yes	272 (50%)	156 (45%)	116 (60%)	
HTN				0.636
No	217 (40%)	137 (39%)	80 (41%)	
Yes	324 (60%)	211 (61%)	113 (59%)	
DM				0.541
No	376 (70%)	245 (70%)	131 (68%)	
Yes	165 (30%)	103 (30%)	62 (32%)	
CAD				0.438
No	495 (91%)	316 (91%)	179 (93%)	
Yes	46 (8.5%)	32 (9.2%)	14 (7.3%)	
Lesion Location				0.062
Basal ganglia or lateral ventricles	237 (44%)	142 (41%)	95 (49%)	
Brain stem or Cerebellum	152 (28%)	106 (30%)	46 (24%)	
Thalamus	15 (2.8%)	12 (3.4%)	3 (1.6%)	
cerebral lobe	35 (6.5%)	27 (7.8%)	8 (4.1%)	
Multiple infarction	102 (19%)	61 (18%)	41 (21%)	
Stroke Location				0.358
Left	339 (63%)	213 (61%)	126 (65%)	
Right	180 (33%)	118 (34%)	62 (32%)	
Both	22 (4.1%)	17 (4.9%)	5 (2.6%)	
NIHSS	3.00 (1.00, 4.00)	2.00 (1.00, 3.00)	4.00 (3.00, 5.00)	<0.001
Age(years)	63.00 (55.00, 70.00)	62.00 (55.00, 70.00)	63.00 (56.00, 70.00)	0.916
MoCA	25.00 (21.00, 26.00)	25.00 (24.00, 26.00)	21.00 (20.00, 24.00)	<0.001
BMI	22.44 (18.63, 27.09)	22.16 (18.34, 26.72)	23.15 (19.64, 27.74)	0.055
WBC(×10^9/L)	6.40 (5.30, 7.70)	6.40 (5.20, 7.70)	6.40 (5.53, 7.90)	0.329
Lym(×10^9/L)	2.53 (1.97, 3.36)	2.54 (1.98, 3.38)	2.52 (1.91, 3.32)	0.473
Mon(×10^9/L)	0.53 (0.44, 0.65)	0.52 (0.43, 0.65)	0.55 (0.46, 0.65)	0.302
Neu(×10^9/L)	5.94 (4.77, 6.89)	6.02 (5.16, 6.89)	5.70 (3.42, 6.86)	0.029
PLT(×10^9/L)	238.76 (194.28, 284.48)	235.97 (194.59, 284.20)	241.47 (192.64, 285.27)	0.462
D-dimer (ng/mL)	125.00 (77.00, 253.00)	112.00 (70.00, 193.50)	178.00 (94.00, 354.00)	<0.001
TG(mmol/L)	1.48 (1.22, 1.75)	1.47 (1.17, 1.82)	1.49 (1.26, 1.72)	0.984
TC(mmol/L)	4.16 (3.47, 4.88)	4.16 (3.51, 4.98)	4.15 (3.44, 4.77)	0.365
LDL(mmol/L)	2.66 (2.22, 3.18)	2.61 (2.19, 3.27)	2.68 (2.27, 3.10)	0.440
Hcy(umol/L)	16.60 (12.90, 26.50)	16.20 (12.90, 25.80)	17.60 (12.90, 27.10)	0.413
UHR	322.13 (238.75, 440.12)	292.17 (228.23, 396.06)	375.92 (281.57, 495.38)	<0.001

^1^n (%); Median (Q1, Q3).

^2^Pearson’s Chi-squared test; Wilcoxon rank sum test.

HTN, hypertension; DM, diabetes mellitus; CAD, Coronary Artery Disease; WBC, white blood cell; Lym, lymphocyte; Mon, monocyte; Neu, neutrophil; PLT, platelet counts; TG, triglycerides; TC, total cholesterol; LDL, low-density lipoprotein; Hcy, homocysteine; UHR, uric acid to HDL ratio.

### 3.2 Association between UA/HDL ratio and PSD

Univariate regression analyses (**Supplementary Table 1**) indicated that sex, smoking, alcohol consumption, NIHSS, MoCA, Neu, D-dimer, and UHR were all positively associated with post-stroke depression in AIS patients. Multivariable logistic regression (**Table 2**) further demonstrated a significant positive association between UHR and PSD. In Model 1, a one-unit increase in UHR was associated with a 0.31% increase in the risk of post-stroke depression (OR = 1.0031, 95% CI: 1.0020–1.0043, P<0.0001). After further adjustment for potential confounders in Model 2, this association remained statistically significant (OR = 1.0023, 95% CI: 1.0007–1.0039, P = 0.0042).

**Table 2:** Multivariable logistic regression analysis of the association between UHR and 90-day PSD.

Exposures	Model1 OR(95%CI), *P*-value	Model2 OR(95%CI), *P*-value
UHR	1.0031(1.0020, 1.0043),<0.0001	1.0023(1.0007, 1.0039), 0.0042
Quartiles		
Q1	Reference	Reference
Q2	1.2317(0.7088, 2.1499),0.4603	1.0884(0.4977, 2.3928),0.8319
Q3	2.4752(1.4709, 4.2255),0.0007	2.2283(1.0747, 4.7143),0.0331
Q4	3.5411(2.1134, 6.0378), <0.0001	2.1707(1.0253, 4.6605), 0.0442
P for trend	<0.0001	0.0113

Model 1 = no covariates were adjusted.

Model 2 = Model 1 + sex, smoking, drinking, NIHSS, MoCA, Neu, D-dimer,were adjusted.

Detailed effect estimates for the association between UHR and 90-day PSD risk in AIS patients are presented in **Supplementary Table S2**. In the fully adjusted model (Model 2), clinically intuitive metrics revealed that each 100-unit increase in UHR was significantly associated with a higher risk of 90-day PSD (OR = 1.2534, 95% CI: 1.0778–1.4702, P = 0.0042). Additionally, a 1-SD increase in UHR corresponded to a notable elevation in 90-day PSD risk (OR = 1.4725, 95% CI: 1.1370–1.9357, P = 0.0042). These results consistently confirm the positive association between UHR and 90-day PSD risk, with effect sizes easily interpretable for clinical practice.

Furthermore, in quartile analyses of UA/HDL, the highest quartile (Q4) was significantly associated with an increased risk of post-stroke depression in fully adjusted Model 2 (OR = 2.1707, 95% CI: 1.0253–4.6605, P = 0.0442). The trend test (P = 0.013) indicated a significant dose-response relationship, with higher UHR quartiles corresponding to progressively greater risk of post-stroke depression.

Sensitivity analyses were performed using two stricter HAMD cut-off values (HAMD >10 and >13), with detailed results presented in **Supplementary Tables S3** and **S4**. In unadjusted models, the association between UHR and 90-day PSD remained statistically significant across both thresholds. After full adjustment (Model 2), notable differences emerged by cut-off: at the HAMD >13 threshold, the continuous UHR variable no longer reached statistical significance (adjusted OR = 1.0004 per unit; 95% CI 0.9990–1.0018; P = 0.55), yet critically, the direction of the effect and its overall trend remained consistent with the primary analysis; in contrast, at the HAMD >10 threshold, the significant association between continuous UHR and 90-day PSD persisted (adjusted OR = 1.0024 per unit; 95% CI 1.0009–1.0039; P = 0.0025) — matching the results of the primary analysis using the HAMD >7 cut-off — and the effect of the highest UHR quartile remained evident. Together, these results validate the robustness of the main findings regarding UHR and PSD risk, even when applying stricter diagnostic criteria for PSD.

### 3.3 Evaluation of nonlinear relationships

**Figure 2:**
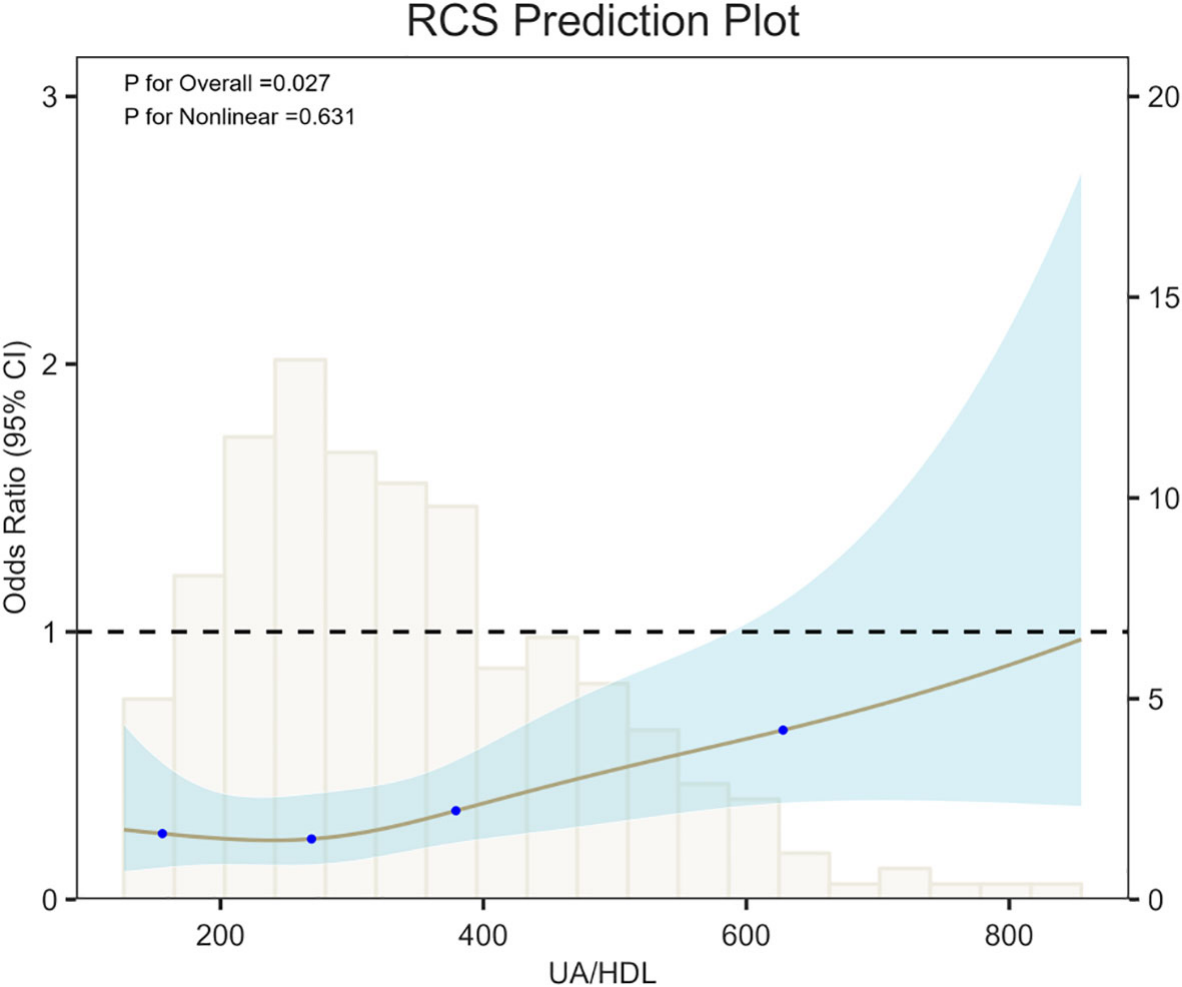
RCS showing the association between UHR and 90-day PSD.

Restricted cubic spline (RCS) analysis (**Figure 2**) was performed with 4 knots, set at the 5th, 35th, 65th, and 95th percentiles of the data (corresponding values: 154.0426, 268.8889, 380.3448, and 628).This analysis demonstrated that increasing UHR were associated with a continuous rise in the risk of 90-day PSD in AIS patients, showing a significant positive trend (P for overall = 0.027). A likelihood ratio test for nonlinearity yielded a χ² value of 0.9217 (df=2, P = 0.631), indicating no significant nonlinear relationship; thus, the association between UHR and 90-day post-stroke depression appears to be linear.

### 3.4 Subgroup analysis

**Figure 3:**
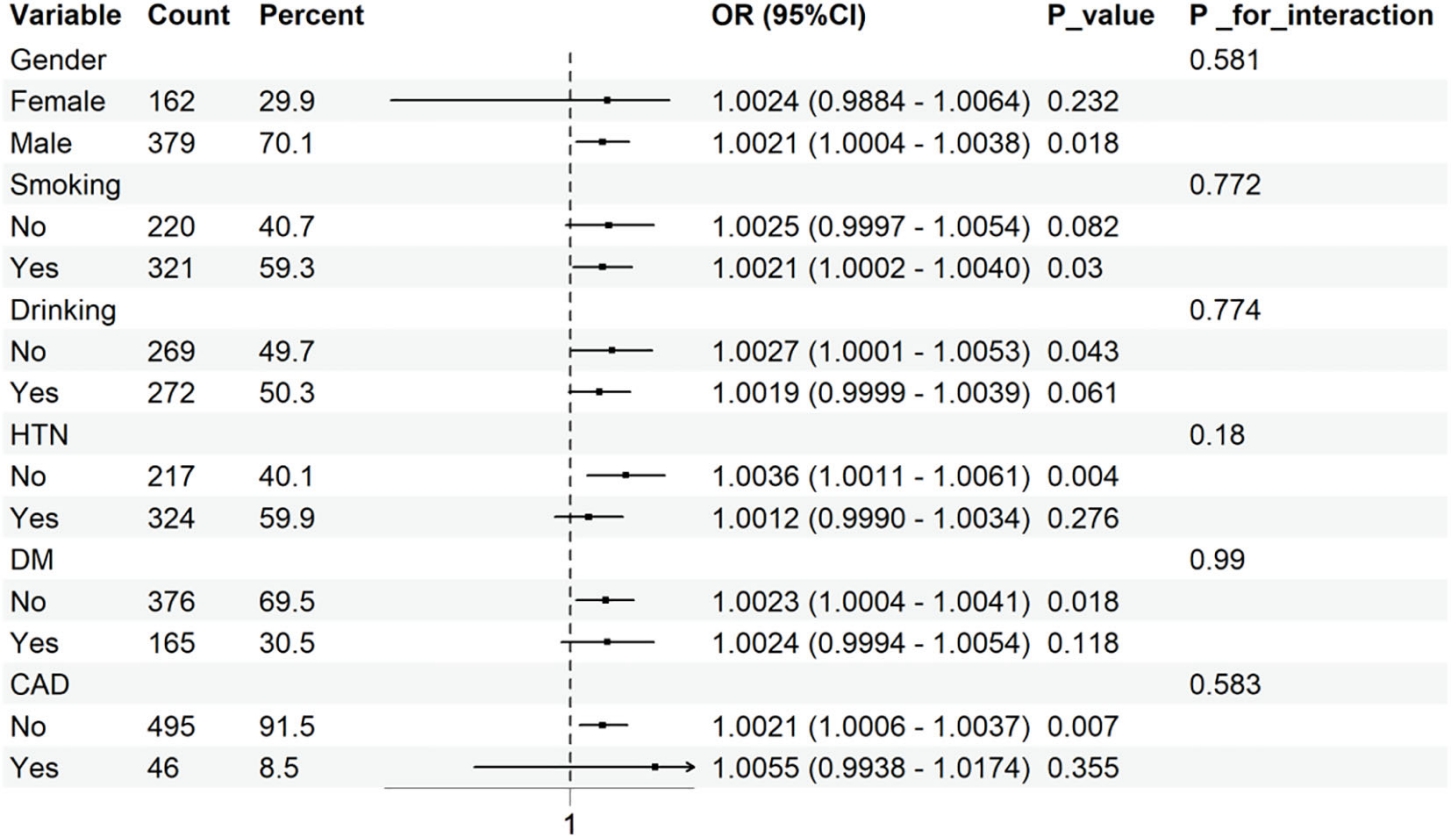
Subgroup analysis for the association between UHR and 90-day PSD.

As illustrated in **Figure 3**, no significant interactions were observed between UHR and any of these subgroups (all interaction P>0.05).

## 4 Discussion

This study is the first to demonstrate in an AIS cohort that elevated UHR at admission is linearly and positively associated with 90-day PSD. After adjusting for potential confounders, including cognitive function and stroke severity, UHR remained an independent predictor of PSD risk. These findings provide novel clinical evidence supporting the “oxidative-antioxidative imbalance” hypothesis and suggest that UHR could serve as a cost-effective, easily measurable, and mechanism-based tool for early PSD risk assessment in the acute phase of AIS.

Although elevated UHR has been linked to various metabolic-inflammatory conditions, including myocardial infarction (21), sarcopenia (22), chronic obstructive pulmonary disease (23), diabetic nephropathy (24), chronic kidney disease (25), coronary artery disease (26), and stroke risk (27), its role in mood disorders—particularly PSD—has not been investigated. A cross-sectional study first reported that, among US adults, higher UHR was significantly associated with increased depression risk, with individuals in the highest UHR quartile having a 42% greater likelihood of depression than those in the lowest quartile (28). However, a separate study in middle-aged and older Chinese adults reported a significant negative association between depressive symptoms and UHR (14). These discrepancies may arise from differences in sample characteristics, racial or ethnic backgrounds, the depression assessment tools employed, and the set of confounders adjusted for. Importantly, neither study examined AIS populations, where acute post-stroke inflammation and lipid remodeling may strengthen the link between UHR and mood disorders, limiting the extrapolation of results from general population studies. Notably, our findings align with recent emergency-department evidence showing that altered serum uric acid levels correlate with acute stroke severity and short-term outcomes, and extend the clinical relevance of UA—already available in most acute-care panels—to early PSD risk stratification (29, 30).

Nevertheless, the underlying mechanisms by which UHR contributes to PSD remain unclear. Within the context of the acute pathophysiological changes following stroke, the contributions of UA and HDL can be considered via distinct pathways. During acute ischemia-reperfusion following stroke, xanthine oxidase is activated, catalyzing the conversion of hypoxanthine to UA and generating a burst of reactive oxygen species (ROS) (31). When UA exceeds its physiological antioxidant threshold, it may induce pathological damage through multiple mechanisms: (1) overproduction of ROS via the mitochondrial respiratory chain and NADPH oxidases (NOXs) (32);(2) activation of the NF-κB pathway, resulting in upregulation of pro-inflammatory cytokines (33);(3) assembly of the NLRP3 inflammasome, triggering IL-1β-mediated inflammatory responses (34, 35);and (4) inhibition of cerebral endothelial nitric oxide synthase, reducing hippocampal perfusion and disrupting depression-related neural circuits (36). Excessive ROS further compromise blood-brain barrier integrity, promote infiltration of peripheral inflammatory mediators into the CNS, ultimately damaging the prefrontal–hippocampal circuitry and triggering depressive-like behaviors. Clinical evidence also indicates that elevated UA levels at admission in elderly AIS patients are closely linked to the development of severe PSD within three months post-stroke (7). In summary, UA may contribute to PSD pathogenesis by promoting free radical production, inducing inflammatory responses, and disrupting nitric oxide (NO) metabolism, thereby generating pro-oxidative stress and causing central nervous system injury (37).

In contrast to the pro-damaging effects of UA, HDL exhibits well-established neuroprotective functions under physiological conditions, mainly via three mechanisms: First, HDL facilitates reverse cholesterol transport, removing excess cholesterol from neurons and surrounding tissues, thereby preserving membrane integrity and functional stability (38);Second, HDL inhibits NADPH oxidase activity, reducing ROS generation and protecting neurons from oxidative stress-induced damage (39);Third, through apolipoprotein A-I, HDL suppresses the release of pro-inflammatory cytokines and inhibits infiltration of inflammatory cells, mitigating neuroinflammation (40). Furthermore, as a carrier of sphingosine-1-phosphate (S1P), reduced HDL levels impair S1P transport and delivery, weakening neuroprotective signaling and indirectly facilitating the development of PSD (41). Clinical evidence further supports a strong link between HDL and depression, with low HDL levels associated with significantly increased depression risk (42). Studies in patients with major depression have shown decreased serum HDL, impaired reverse cholesterol transport, and significant correlations between HDL and immune-inflammatory markers (43). More directly, a recent clinical study demonstrated that low HDL levels are independently associated with the occurrence of PSD at three months post-stroke (12). Collectively, these findings suggest that HDL may contribute to PSD pathogenesis through modulation of inflammatory and immune responses.

UHR simultaneously captures the pro-oxidative burden of UA and the antioxidative reserve of HDL. An elevated UHR indicates that pro-oxidative forces surpass anti-inflammatory defenses, representing a “tipping point” in metabolic-inflammatory imbalance. This imbalance both damages neurons through the ROS–inflammasome–cytokine axis and impairs neural repair due to HDL functional deficits, synergistically increasing PSD risk. The linear dose–response relationship observed in this study further supports this integrated model: as UHR increases, the metabolic-inflammatory imbalance intensifies, accompanied by a corresponding rise in PSD risk.

In routine stroke unit assessments, UA and HDL are standard admission tests, allowing UHR to be calculated directly from existing data at no extra cost. This immediacy makes UHR a practical tool for early identification of patients at risk for PSD. Patients with elevated UHR could be prioritized for early psychological screening and behavioral interventions, enabling targeted management. Future prospective studies could evaluate whether UHR-based risk stratification allows precise identification of high-risk individuals, optimizes intervention timing, and ultimately reduces the incidence of PSD.

This study offers several notable strengths: First, it represents the first cohort study to examine the association between UHR and PSD in an AIS population, addressing a gap in the current literature. Second, the robustness of this association was confirmed via RCS modeling and subgroup analyses. Finally, outcomes were assessed using the standardized the HAMD, ensuring both reliability and comparability of the findings.

Nevertheless, this study has several limitations: First, the single-center retrospective design may introduce selection bias and precludes complete control over residual confounding variables; Second, only baseline UHR levels were assessed, without longitudinal monitoring at multiple post-stroke time points, limiting the ability to establish temporal relationships between UHR dynamics and PSD development. Third, data on post-stroke interventions—antidepressant initiation, rehabilitation intensity, and psychosocial support—were unavailable, any of which could modify PSD risk. Fourth, renal function, urate-lowering drugs (allopurinol/febuxostat), diuretics, and statins, all of which influence uric acid or HDL levels, were not recorded and may confound the observed association. Therefore, future research should focus on multicenter, large-scale prospective cohort studies to systematically capture these unmeasured factors, validate UHR as a predictor of PSD and to establish individualized thresholds based on its dynamic changes across different post-stroke phases, thereby enhancing clinical applicability. Furthermore, serial UHR measurements after admission may offer incremental predictive value over a single baseline value and warrant exploration in future longitudinal studies.

## 5 Conclusion

As an integrated marker of the oxidative–antioxidative system, UHR independently and linearly predicts the 90-day risk of PSD in AIS patients, offering a potentially simple and practical tool for precise identification of high-risk individuals.

## Data Availability

The raw data supporting the conclusions of this article will be made available by the authors, without undue reservation.
