# Supplementary material for: Admission uric acid to HDL-C ratio predicts 90-day post-stroke depression in acute ischemic stroke
Source: Front Psychiatry. 2025 Nov 3;16:1693156. doi: 10.3389/fpsyt.2025.1693156 (PMC12620973; doi:10.3389/fpsyt.2025.1693156)
Supplement: Supplementary file 1 [file Table1.docx]

Catalogue

[◼ **Table S1** Univariate logistic regression analysis 2](#_Toc210144202)

[◼ **Table S2** Multivariable Logistic Regression of UHR and 90-day PSD by Scaling Metrics 4](#_Toc210144203)

[◼ **Table S3** Multivariable Logistic Regression Analysis of the Association Between UHR and 90-day PSD(HMHD>10). 4](#_Toc210144204)

[◼ **Table S4** Multivariable Logistic Regression Analysis of the Association Between UHR and 90-day PSD(HMHD>13). 4](#_Toc210144205)

- **Table S1** Univariate logistic regression analysis

| **Characteristic** | **OR** | **95% CI** | **p-value** |
| --- | --- | --- | --- |
| NIHSS | 2.133 | 1.855, 2.480 | <0.001 |
| Age | 0.9989 | 0.9825, 1.016 | 0.895 |
| MoCA | 0.5899 | 0.5351, 0.6460 | <0.001 |
| BMI | 1.025 | 0.9982, 1.052 | 0.068 |
| WBC | 1.051 | 0.9835, 1.124 | 0.139 |
| Lym | 0.8793 | 0.7378, 1.044 | 0.146 |
| Mon | 1.635 | 0.5614, 4.761 | 0.366 |
| Neu | 0.8727 | 0.7950, 0.9573 | 0.004 |
| PLT | 1.001 | 0.9987, 1.004 | 0.338 |
| D-dimer | 1.001 | 1.000, 1.001 | 0.003 |
| TG | 0.9318 | 0.6538, 1.307 | 0.687 |
| TC | 0.8963 | 0.7547, 1.061 | 0.207 |
| LDL | 1.032 | 0.8253, 1.290 | 0.781 |
| Hcy | 1.004 | 0.9963, 1.013 | 0.284 |
| UA/HDL | 1.003 | 1.002, 1.004 | <0.001 |
| Sex |  |  |  |
| Female | — | — |  |
| Male | 3.155 | 2.052, 4.971 | <0.001 |
| Education |  |  |  |
| illiteracy | — | — |  |
| Primary school | 1.293 | 0.7651, 2.216 | 0.342 |
| junior high school | 1.067 | 0.6347, 1.819 | 0.809 |
| senior high school and above | 1.017 | 0.4585, 2.203 | 0.966 |
| Smoking |  |  |  |
| No | — | — |  |
| Yes | 1.955 | 1.353, 2.848 | <0.001 |
| Drinking |  |  |  |
| No | — | — |  |
| Yes | 1.854 | 1.299, 2.657 | <0.001 |
| HTN |  |  |  |
| No | — | — |  |
| Yes | 0.9171 | 0.6414, 1.314 | 0.636 |
| DM |  |  |  |
| No | — | — |  |
| Yes | 1.126 | 0.7680, 1.643 | 0.541 |
| CAD |  |  |  |
| No | — | — |  |
| Yes | 0.7723 | 0.3901, 1.458 | 0.439 |
| LesionLocation |  |  |  |
| Basal ganglia or lateral ventricles | — | — |  |
| Brain stem or Cerebellum | 0.6487 | 0.4187, 0.9963 | 0.050 |
| Thalamus | 0.3737 | 0.0835, 1.214 | 0.135 |
| cerebral lobe | 0.4429 | 0.1814, 0.9753 | 0.055 |
| Multiple infarction | 1.005 | 0.6234, 1.610 | 0.985 |
| StrokeLocation |  |  |  |
| Left | — | — |  |
| Right | 0.8882 | 0.6067, 1.294 | 0.539 |
| Both | 0.4972 | 0.1603, 1.292 | 0.180 |

- **Table S2** Multivariable Logistic Regression of UHR and 90-day PSD by Scaling Metrics

| **Exposures** | **Model1**  **OR(95%CI),*P*-value** | **Model2**  **OR(95%CI),*P*-value** |
| --- | --- | --- |
| UHR |  |  |
| Per 1-unit increase | 1.0031(1.0020, 1.0043),<0.0001 | 1.0023(1.0007, 1.0039), 0.0042 |
| Per 100-unit increase | 1.3661(1.2205, 1.5403), <0.0001 | 1.2534(1.0778, 1.4702),0.0042 |
| Per 1-SD increase | 1.7067(1.4070, 2.0965), <0.0001 | 1.4725(1.1370, 1.9357), 0.0042 |

*Model 1 = no covariates were adjusted.

Model 2 = Model 1 + sex，smoking, drinking, NIHSS, MoCA, Neu, D-dimer,were adjusted.

- **Table S3** Multivariable Logistic Regression Analysis of the Association Between UHR and 90-day PSD(HMHD>10).

| **Exposures** | **Model1**  **OR(95%CI),*P*-value** | **Model2**  **OR(95%CI),*P*-value** |
| --- | --- | --- |
| UHR | 1.0033(1.0021, 1.0045),<0.0001 | 1.0024(1.0009, 1.0039), 0.0025 |
| Quartiles |  |  |
| Q1 | Reference | Reference |
| Q2 | 1.1020(0.6160, 1.9765),0.7430 | 0.9268(0.4202, 2.0499),0.8503 |
| Q3 | 2.3418(1.3709, 4.0650),0.0021 | 2.0118(0.9701, 4.2566),0.0630 |
| Q4 | 3.8004(2.2451, 6.5662), <0.0001 | 2.3678(1.1318, 5.0440), 0.0233 |
| P for trend | <0.0001 | 0.0039 |

*Model 1 = no covariates were adjusted.

Model 2 = Model 1 + sex，smoking, drinking, NIHSS, MoCA, Neu, D-dimer,were adjusted.

- **Table S4** Multivariable Logistic Regression Analysis of the Association Between UHR and 90-day PSD(HMHD>13).

| **Exposures** | **Model1**  **OR(95%CI),*P*-value** | **Model2**  **OR(95%CI),*P*-value** |
| --- | --- | --- |
| UHR | 1.0020(1.0009, 1.0031),0.0003 | 1.0004(0.9990, 1.0018), 0.5500 |
| Quartiles |  |  |
| Q1 | Reference | Reference |
| Q2 | 1.2147(0.6687, 2.2194),0.5234 | 1.1857(0.5560, 2.5567),0.6604 |
| Q3 | 1.9366(1.1039, 3.4520),0.0226 | 1.4394(0.7006, 3.0037),0.3250 |
| Q4 | 2.8698(1.6629, 5.0585), 0.0002 | 1.4192(0.6911, 2.9462), 0.3423 |
| P for trend | <0.0001 | 0.2968 |

*Model 1 = no covariates were adjusted.

Model 2 = Model 1 + sex，smoking, drinking, NIHSS, MoCA, Neu, D-dimer,were adjusted.
